# Transcriptomic Response of the Ovarian Follicle Complex in Post-Vitellogenic Rainbow Trout to 17α,20β-Dihdroxy-4-pregnen-3-one In Vitro

**DOI:** 10.3390/ijms252312683

**Published:** 2024-11-26

**Authors:** Hao Ma, Guangtu Gao, Yniv Palti, Vibha Tripathi, Jill E. Birkett, Gregory M. Weber

**Affiliations:** 1 US Department of Agriculture, Agricultural Research Service, National Center for Cool and Cold Water Aquaculture, 11861 Leetown Road, Kearneysville, WV 25430, USAyniv.palti@usda.gov (Y.P.); vibha.tripathi2@usda.gov (V.T.); jill.birkett@usda.gov (J.E.B.); 2 US Department of Agriculture, Agricultural Research Service, National Animal Disease Center, Ruminant Disease and Immunology Research Unit, Ames, IA 50010, USA; hao.ma@usda.gov

**Keywords:** progestin, meiosis, maturational competence, signaling, teleost

## Abstract

Gonadotropins and progestins are the primary regulators of follicle maturation and ovulation in fish, and they require complex communication among the oocyte and somatic cells of the follicle. The major progestin and the maturation-inducing hormone in salmonids is 17α,20β-dihdroxy-4-pregnen-3-one (17,20βP), and traditional nuclear receptors and membrane steroid receptors for the progestin have been identified within the follicle. Herein, RNA-seq was used to conduct a comprehensive survey of changes in gene expression throughout the intact follicle in response to in vitro treatment with these hormones to provide a foundation for understanding the coordination of their actions in regulating follicle maturation and preparation for ovulation. A total of 5292 differentially expressed genes were identified from our transcriptome sequencing datasets comparing four treatments: fresh tissue; untreated control; 17,20βP-treated; and salmon pituitary homogenate-treated follicles. Extensive overlap in affected genes suggests many gonadotropin actions leading to the acquisition of maturational and ovulatory competence are mediated in part by gonadotropin induction of 17,20βP synthesis. KEGG analysis identified signaling pathways, including MAPK, TGFβ, FoxO, and Wnt signaling pathways, among the most significantly enriched pathways altered by 17,20βP treatment, suggesting pervasive influences of 17,20βP on actions of other endocrine and paracrine factors in the follicle complex.

## 1. Introduction

Captive fish often display reproductive dysfunction associated with follicle maturation and ovulation [1]. Ovarian follicle maturation (OFM) and ovulation in teleost fish are overlapping processes primarily regulated by the gonadotropin (GTH) luteinizing hormone (Lh) and progestin steroids, including the maturation-inducing hormone (MIH) (see reviews [2,3,4,5,6,7]). Estradiol-17β (E2) has also been implicated in the maintenance of meiotic arrest in the fish oocyte [8] and has been shown to antagonize GTH-induced oocyte maturation and MIH production but not MIH-induced meiosis resumption in rainbow trout [9,10,11]. The major progestin and MIH in salmonids, including rainbow trout (*Oncorhynchus mykiss*), is 17α,20β-dihdroxy-4-pregnen-3-one (17,20βP) [12]. Gonadotropin-dependent ovarian follicle maturation itself also takes place in overlapping stages in most fishes. In the first stage, oocyte maturational competence (OMC), the somatic follicle cells acquire the ability to produce the MIH, and the oocyte acquires the ability to respond to the MIH. In the second stage, oocyte maturation, the follicle cells produce MIH, which in turn activates the maturation-promoting factor responsible for releasing the oocyte from meiotic arrest [12,13]. The oocyte must also undergo maturational changes, including changes in the cytoplasm, to acquire full oocyte developmental competence, which is the ability of the oocyte to develop into a functional embryo [3,14,15].

After the follicle develops the ability to produce the MIH, it must develop competence to respond to hormonal signals to induce ovulation and then respond to hormones to undergo ovulation [2,16]. The GTH, Lh, is the primary regulator of MIH production and meiosis resumption in rainbow trout [17]. Luteinizing hormones and progestins also act as mediators of ovulatory processes, which lead to the expulsion of an egg that is arrested at metaphase II and free of somatic follicle cells [2,16]. In rainbow trout, 17,20βP can induce oocyte maturation earlier than it is able to induce oocyte developmental competence or ovulation [18,19]. The stage at which GTH and progestins can induce both OFM and ovulation and produce a developmentally competent oocyte is referred to as follicular maturational competence (FMC) [14,15,19]. During follicle maturation, the oocyte nucleus or germinal vesicle (GV) migrates to the periphery of the oocyte, after which the nuclear membrane disintegrates (germinal vesicle breakdown; GVBD) as the oocyte exits meiotic arrest at the first meiotic prophase [6]. Maturational competence of the follicle complex is acquired as the GV migrates to the periphery of the oocyte [10,20]. Although Lh is the primary regulator of OFM in salmonids, the involvement of the follicle-stimulating hormone (Fsh) is also likely. An increase in the Fsh receptor (fshr) expression during GVM may suggest a role of Fsh in the acquisition of FMC [14].

Despite the central roles of GTHs and the progestins in regulating OFM and ovulation in teleosts being well established, many aspects of their mechanisms of action remain unresolved. Direct actions of GTHs are limited to the somatic follicle cells as GTH receptors are present on theca and granulosa cells but not on the oocyte membrane [21,22]. Progestins can act through nuclear progestin receptors (nPRs), membrane progestin receptors (mPRs) and progesterone receptor membrane components (Pgrmcs), and crosstalk between the different receptor types is evident [23,24]. Whereas the MIH has been shown to induce meiosis resumption through non-genomic pathways, and progestins, which may also be the MIH, have been shown to induce ovulation through genomic action, other genomic actions of progestins in post-vitellogenic follicles requiring changes in gene expression remain poorly characterized [25,26,27,28,29,30]. The primary progestin in salmonids is 17,20βP, but in other teleosts, multiple progestins are present and exhibit different affinities for the nPRs and mPRs, suggesting differential regulation of processes including oocyte maturation and ovulation [25,26]. In the spotted sea trout (*Cynoscion nebulosus*), 17α,20β,21-trihdroxy-4-pregnen-3-one (20βS) is the putative MIH and has a higher binding affinity for the mPR than 17,20βP, whereas 17,20βP is more potent at inducing ovulation and binds to the nPR with greater affinity than 20βS [25,26,27,31,32].

As part of efforts to better understand processes involved in OFM and ovulation in teleosts, changes in global gene expression as the follicle progresses through OFM and ovulation naturally (e.g., [30,33,34,35,36]) or in response to in vivo hormone treatments with GTHs (e.g., [37,38]) have been investigated. A large set of differentially expressed genes representing a wide array of functions were identified using RNA-seq to compare transcriptomes of follicles collected after oocyte maturation but before ovulation from wild type and nPR-knockout Zebrafish supporting extensive actions transmitted through the nPR during later stages of OFM [30]. Over a thousand transcripts within diverse biological pathways have been identified as changing during the transition from fully grown post-vitellogenic follicles through ovulation in rainbow trout alone [39,40,41,42]. Direct actions of the endocrine and paracrine factors regulating these events have also been extensively investigated, including the use of in vitro bioassay approaches; however, few of these studies have included global mRNA analysis.

The broader objective of the current study was to provide a global view of the role of progestins in regulating OFM within the follicle complex to provide direction for future, more in-depth investigations of progestin actions in specific cell types of the complex and through specific receptors throughout the complex. The specific objective of the present study was to identify the transcriptomic response of the follicle complex to 17,20βP at the onset of OMC. Treatment with salmon pituitary homogenate (SPH) was included in the study as a preliminary comparison of this response to that of GTHs. The freshly excised tissues were also included in the study primarily to document the transcriptome signature of the follicles at the start of the study and facilitate comparisons with in vivo developmental studies. Comparing the transcriptomes of the freshly excised tissues with controls after 24 h of incubation may also provide some insight into the endogenous regulatory environment from which the follicles are released for incubation, as well as other perturbations of the culture protocol. In the present study, we characterized the in vitro effects of the MIH on the transcriptome of the follicle complex during late-GV migration (GVM) using RNA-seq analysis of polyadenylated mRNA enriched libraries. Transcriptomes of intact follicles incubated with 17,20βP for ~24 h were compared to those of follicles incubated ~24 h with SPH as a source of GTHs, follicles incubated without hormone for ~24 h, and follicles freshly excised from the female. The intact follicles include the transcriptomes of the oocyte, theca, granulosa, and stromal cells.

The study identified 32,000 sequences, with 5292 sequences among diverse biological processes differentially expressed in response to the treatments. A majority of the differentially expressed genes (DEGs), 3701, were found in the comparison between follicles incubated with and without 17,20βP. Extensive overlap among DEGs in response to SPH and 17,20βP suggests many gonadotropin actions leading to the acquisition of maturational and ovulatory competence are mediated in part by gonadotropin induction of 17,20βP synthesis. KEGG pathway analysis identified several signal transduction pathways among the most significantly enriched pathways in response to hormone treatments, and therefore, our discussion is focused on the effects of 17,20βP and SPH treatment on growth factor signaling pathways. The MAPK, TGFβ, FoxO, and Wnt signaling pathways are among the most significantly enriched KEGG pathways altered by 17,20βP treatment, suggesting pervasive influences of 17,20βP on actions of other endocrine and paracrine factors in the follicle complex. This study provides a basis for elucidating the early actions of progestins in preparing the fully grown post-vitellogenic follicle for maturation and ovulation.

## 2. Results

### 2.1. Assessment of Follicle Competence

The rapid and dynamic changes taking place in the follicle as it progresses through OFM make it difficult to select follicles at the same stage of development for use in the study of hormone actions. Sensitivity to endocrine factors, including the MIH and the follicle transcriptome, are in flux. Selection criteria were used to characterize and synchronize the developmental stage of the follicles. Follicles expected to have had minimal in vivo exposure to MIH were preferred to better identify genes responsive to the steroid. Nine fish were first selected based on having oocytes that were near late-GVM and did not exhibit clearing of the ooplasm. Second, the tissues were evaluated for maturational competence in vitro. Tissues that responded with 80% or more of the oocytes completing GVBD within 96 h of incubation in response to 290 nM 17,20βP, but no oocytes completing GVBD without hormone treatment were selected (Table 1). Fish # 1, #5, #6, # 8, and #9 met these criteria. Some competence to respond to SPH to induce resumption of meiosis was also preferred. Fish #1, #5, #6, and #8 best met these criteria, but follicle survival in culture was low for fish #8. We chose fish #5 and #6 for RNA-seq analysis based on all criteria and selected fish #1 over fish #8 and #9, preferring a similar response to SPH in terms of GVBD induction. Based on casual observation, after 24 h of incubation, most of the oocytes treated with 17,20βP showed the initiation of ooplasm clearing, and the GV were late in migration or at the periphery of the oocyte.

### 2.2. RNA-Seq Read Alignments

**Figure 1:**
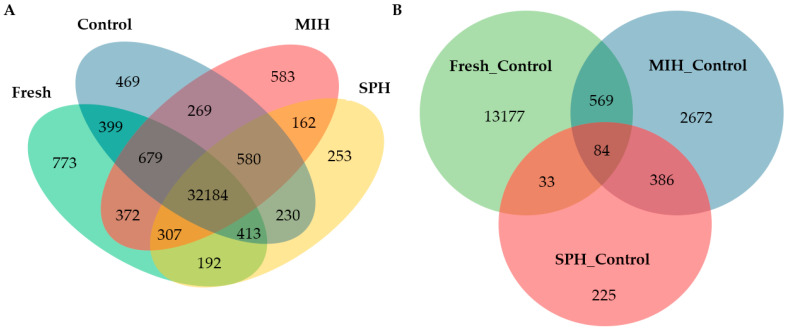
Venn diagram showing the distribution of all mapped and differentially expressed genes: (**A**) Number of transcripts mapped to rainbow trout reference transcriptome for fresh tissue, untreated control, MIH treated, and SPH treated samples. (**B**) Venn diagram depicting commonalities of differentially expressed transcripts identified by both DESeq2 and edgeR programs for MIH_control, SPH_control, and Fresh_control comparisons (FDR < 0.05).

RNA sequencing with Illumina HiSeq 2000 platform generated 18.29–25.66, 23.85–36.41, 22.42–34.14, and 24.24–26.16 million raw reads from the samples for the control, fresh, MIH treated, and SPH treated follicles, respectively (Table 2). The results indicated that 74.29% of the raw reads were uniquely aligned to the rainbow trout reference genome, and 14.45–18.27% of the raw reads were aligned to multiple locations. The fact that about 16.50% of the aligned reads were mapped to multiple locations reflects the complexity of rainbow trout genome evolution [43,44]. A total of 39,027–41,906 reference transcripts were mapped by the sequences derived from the 12 libraries, among which 32,184 were shared by the four treatment groups, and only 253 to 773 transcripts were unique to individual groups (Figure 1A).

### 2.3. Differentially Expressed Genes Within and Between Treatment Comparisons

**Figure 2:**
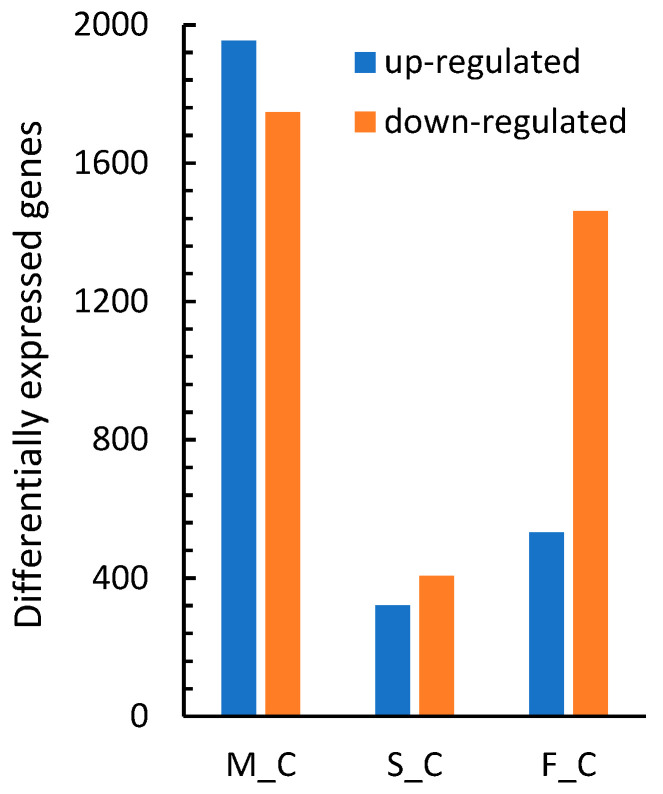
Upregulated and downregulated differentially expressed genes identified by both DESeq2 and edgeR programs for MIH_control (M_C), SPH_control (S_C), and Fresh_control (F_C) comparisons (FDR < 0.05).

Transcriptome responses between the M_C, S_C, and F_C comparisons (Supplemental Tables S2, S3, and S4, respectively, and combined Table S5) were also compared. A total of 5292 differentially expressed genes (DEGs) were identified by both DESeq2 and edgeR programs among the three pairs of comparisons (Figure 1B). The M_C comparison yielded the greatest number of DEGs, with 1954 upregulated and 1747 downregulated genes (Figure 2). The S_C comparison yielded 321 upregulated and 407 downregulated genes, and the F_C comparison yielded 532 upregulated genes and 1461 downregulated genes. The Venn diagram in Figure 1B indicates that the M_C and S_C comparisons shared 470 DEGs or about 65% of the S_C DEGs. Among genes that were differentially expressed in both the M_C and S_C comparisons, 48 were regulated in opposite directions (Supplemental Table S6). The F_C comparison had 1317 specific DEGs and 676 shared with M_C or S_C comparisons.

### 2.4. Gene Set Enrichment Analysis of DEGs

The gene set enrichment analysis (GSEA) methods used are based on enrichment analysis of Gene Ontology (GO) and the Kyoto Encyclopedia of Genes and Genomes (KEGG). The Entrez IDs for all DEGs used for analysis with DAVID are listed in Table S6. In converting the IDs to the most recent version of the annotated rainbow trout reference genome, we began from 5292 genes and removed 275 to get 4917 Entrez IDs (since 100 IDs were discontinued and 175 IDs were duplicated). Enriched GO terms for *biological process* and *molecular function* identified among the three comparisons are shown in Table 3 and Table 4, respectively. The enriched GO terms for a *biological process* for M_C fell under four first-level terms: *biological regulation* (GO:0065007), *cellular process* (GO:0009987), *localization* (GO:0051179), and *response to stimulus* (GO:0050896). Most of the enriched GO terms for S_C were also found for M_C. Even the genes in the enriched GO terms for S_C that were not shared with M_C were mostly DEGs for M_C, including the three DEGs in the *female gamete generation* (GO:0007292). The genes *Id2 protein* (100136777), *eukaryotic peptide chain release factor subunit 1-like* (110537225), and *nuclear cap-binding protein subunit 1* (110488875) in *negative regulation of gene expression* (GO:0010629) were the only genes in S_C enriched GO terms that were not differentially expressed in M_C enriched GO terms. All but one of the first level enriched GO terms in the F_C comparison, *immune system process* (GO:0002376), were shared with the hormone treatments. The overlap is consistent with the control treatment tissues being removed from endogenous hormone exposure. However, lower-level enriched GO terms unique to this comparison, such as *phagocytosis* (GO:0006909), *response to stress* (GO:0006905), and *defense response* (GO:0006952) may suggest effects of the bioassay protocol at 24 h.

The enriched GO terms for *molecular function* for M_C fell under three first-level terms: *binding* (GO:0005488), *catalytic activity* (GO:0003824), and *molecular function regulator activity* (GO:0098772). Only three enriched GO terms were identified for S_C. *Protein binding* (GO:0005515) and *receptor binding* (GO:0005102) were also enriched for M_C, and both had more representative genes for M_C. Only the GO term *cytokine activity* (GO:0005125) was enriched for S_C but not M_C, although *interleukin-12 beta chain* (100136086) and *macrophage colony-stimulating factor precursor* (100136089) in *cytokine activity* were the only genes that were not differentially expressed in M_C. The enriched GO terms for F_C were also very similar to those for M_C. Most terms under *molecular function regulatory activity* that were enriched for F_C but not enriched for M_C, nevertheless, were similar to terms involving growth factor actions under *binding* that were enriched in M_C but not F_C. Enriched terms for F_C compared with M_C are again consistent with DEGs in the F_C comparison resulting from follicle removal from endogenous hormonal regulation in the control group. Enriched GO terms for molecular function for the F_C comparison do not appear distinctive for aberrations due to culture.

**Figure 3:**
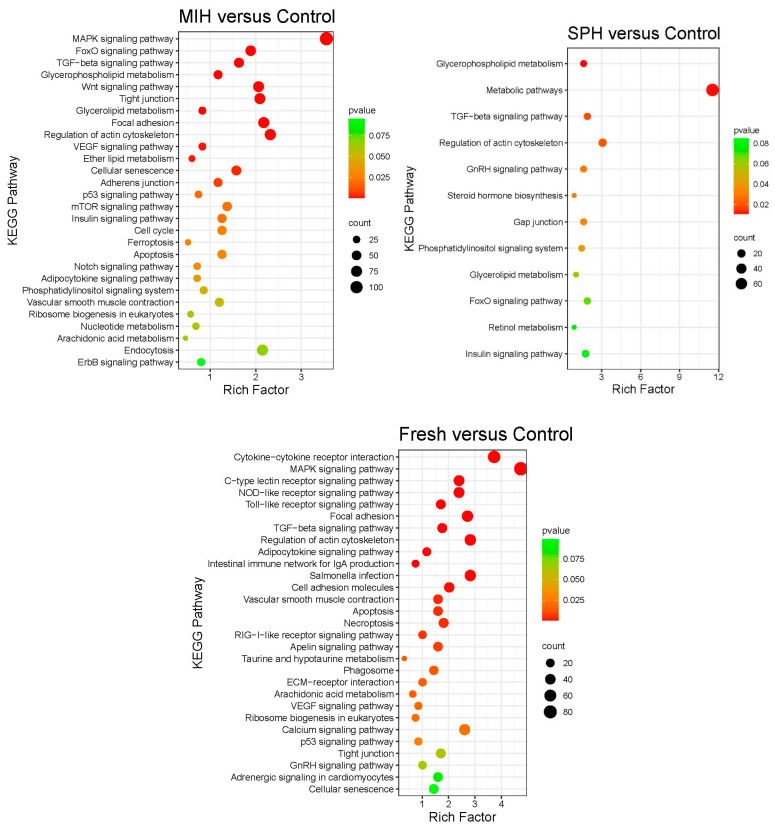
Bubble chart showing enriched KEGG pathways for differentially expressed genes in treatment comparisons. The color and size of the bubbles indicate the range of the *p*-value and the number of DEGs in each pathway.

Similar to what was found with GO term analysis, many more enriched KEGG pathways were identified for M_C than S_C, and about half of the enriched KEGG pathways for S_C are found in the M_C comparison (Figure 3, Supplemental Tables S7 and S8). The M_C comparison also had the most genes in almost all pathways shared with S_C. Even among the five pathways for S_C not shared with M_C, many of the genes in the pathways were DEGs for M_C. In S_C, 51 of the 79 genes in the broad *metabolic pathways*, and about half each in the *gap junction* and *GnRH signaling pathway*, were also differentially expressed in M_C. All but one gene in *steroid biogenesis* and none of the genes in *retinol metabolism* were DEGs for M_C. Considerable overlap in DEGs also exists among different pathways even within the same comparison, as exemplified by all of the genes in *steroid biogenesis* and *retinol metabolism* also being included in *metabolic pathways* for the S_C comparison. Overall, most transcript responses to SPH were also seen with 17,20βP treatment, even for pathways enriched for S_C and not M_C. Treatment with 17,20βP led to the enrichment of a diverse set of signaling pathways, including the *MAPK* (map kinase), *TGFβ* (transforming growth factor-β), *FoxO* (forkhead box transcription factor) *Wnt*, *VEGF* (vascular endothelial growth factor), *Insulin*, and *Notch signaling pathways*. Pathways for regulating cell-to-cell junctions, including *focal adhesion*, *tight junction,* and *adherens junction*, and regulation of programmed cell death, including *apoptosis* and *ferroptosis*, were enriched in response to 17,20βP treatment.

The F_C comparison also shared many enriched KEGG pathways with the other comparisons. However, this comparison also had the highest number of unique pathways, including three of the most statistically significant within the comparison: *cytokine-cytokine receptor interaction*, *C-type lectin receptor signaling pathway*, *NOD-like receptor signaling pathway*, *and toll-like receptor signaling pathway*, each with greater than 30 genes. Pathways specifically enriched in the F_C comparison suggest ways in which the tissues might have been impaired in culture. As an example, pattern recognition receptors (PPRs), including both membrane-bound PPRs, such as toll-like receptors and c-type lectin receptors, and cytoplasmic PPRs, such as NOD-like receptors and RIG-I-like receptors, were enriched, suggesting activation of the innate immune system.

### 2.5. Verification of Differentially Expressed Genes

**Figure 4:**
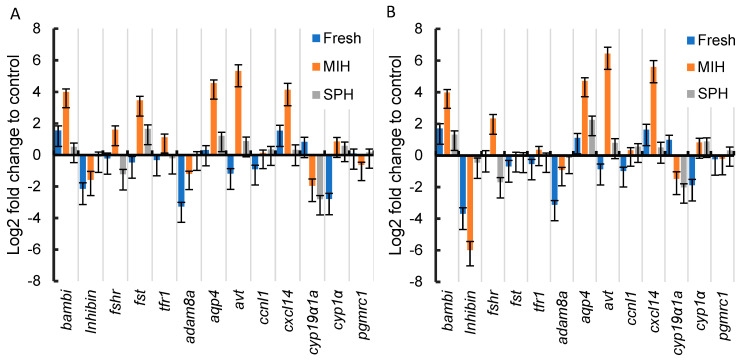
A comparison of 13 genes expressed in fresh, MIH, and SPH treated samples quantified by RT-qPCR and RNA-seq.:(**A**) RT-qPCR quantification of the selected genes. Gene expression value was normalized by *ef1a* and further compared to control as Log2 fold change. (**B**) Log2 fold change in RNA sequencing data was generated by DESeq2. Bars represent means ± SEM.

RT-qPCR was performed to confirm some of the DEGs expressed under different treatments. The 13 DEGs selected for verification were differentially expressed in the MIH_Control comparison and were previously found to be differentially regulated during follicle maturation in rainbow [15,40,45,46]. The relative expressions of the 13 genes quantified by RT-qPCR were highly consistent with the results of RNA sequencing (Figure 4). Spearman’s rank correlation coefficients for Fresh_Control, MIH_Control, and SPH_Control, are 0.9670 (*p*-value = 7.064 × 10^−8^), 0.9286 (*p*-value = 4.607 × 10^−6^), and 0.7527 (*p*-value = 0.0030) respectively.

## 3. Discussion

The transcriptomic response to treatments in the present study reflects the complex processes participating in follicle maturation and ovulation that are initiated once MIH is produced by the post-vitellogenic follicle complex. Progestin production is regulated by GTHs, and therefore, transcriptome responses to increased endogenous 17,20βP would be induced by endogenous increases in GTHs. With the production of progestins being just one action of the GTHs in the post-ovulatory follicle complex, not to mention other pituitary hormones in SPH, treatment with SPH should have a wider genomic influence than 17,20βP. Nevertheless, in the present study, many more DEGs were identified, and GO terms and KEGG pathways enriched in response to 17,20βP treatment than to SPH treatment. The greater transcriptomic response to 17,20βP than SPH is likely due in part to elements of the study protocol. The concentration of SPH used in the study was considerably less effective than the concentration of 17,20βP at inducing GVBD, suggesting lower efficacy of the SPH treatment to elicit progestin-sensitive actions. Purified salmonid GTHs are not commercially available, and salmonids are unusual among fishes in that their GTH receptors do not recognize chorionic gonadotropins [47]. SPH is a crude preparation of acetone-dried pituitary homogenate, and therefore, the magnitude of a response to a single dosage is difficult to evaluate. Furthermore, tissues were sampled at 24 h of incubation for mRNA analysis, providing limited time for SPH treatment to increase 17,20βP synthesis and release and then for the steroid to act, including both stimulation of progestin-sensitive genes and transcriptome responses to metabolites downstream of 17,20βP-sensitive genes. On the other hand, early studies in rainbow trout by Jalabert [18,48] demonstrated that once FMC is induced by GTHs, 17,20βP can induce in vitro oocyte maturation and developmental competence and ovulation, resulting in developmentally competent ovulated oocytes. Therefore, 17,20βP can be expected to induce many of the transcript changes in the follicle complex that take place during OFM, following OMC, given enough time to act. In early FMC follicles, however, as is likely the stage of follicles in the present study, GTH action is still required to achieve full FMC, and a second or much longer treatment with 17,20βP would be required to induce ovulation if at all successful [19]. Genes and pathways modulated specifically by SPH may reflect GTH regulation; however, SPH also contains other pituitary hormones that may have elicited transcriptomic responses.

### 3.1. Follicle Stage

From the end of vitellogenic growth through ovulation, the follicle progresses through dramatic developmental stages with unique transcriptomic signatures and changes in gene expression in response to hormone exposure. Therefore, knowing the stage of the follicles used in a study is critical for interpreting responses. This includes responses to exogenous treatments and the effects of being removed from the endogenous endocrine milieu, as shown in the F_C comparison. The follicles used in the present study were at late GVM and had at least partially achieved OMC at the onset of treatment. They did not complete GVBD until days after the 24 h sampling for transcriptomic analysis. Rainbow trout acquiring OMC are transitioning from the production of E2 to MIH, although plasma MIH levels are often still very low, even in competent fish [20,45]. The extent of migration is not a reliable or precise indicator of OMC or FMC, but late-GVM stage follicles generally are of high OMC but low FMC and are therefore highly sensitive to MIH to induce oocyte maturation but require high concentrations of GTH or MIH to fully complete OFM and ovulation in vitro [10]. Competence was directly assessed based on their ability to complete GVBD in response to both 17,20βP and SPH (Table 1). The follicles selected for the present study had reached OMC before treatment based on MIH and SPH, which induces a resumption of meiosis or oocyte maturation as indicated by GVBD.

Resumption of meiosis in rainbow trout oocytes and separation of the oocyte from the follicle wall can be induced by exogenous MIH before full FMC is acquired. Precocious stimulation of follicles with low FMC with MIH can result in maturing oocytes, but failure to induce ovulation can result in oocytes with low developmental competence [14,15,19]. The three fish selected for RNA-seq analysis all responded well to 17,20βP treatment, completing GVBD in about 80% or greater of the oocytes examined within 96 h, and showed some maturational response to SPH. With adequate dosage and time, GTH can induce oocyte maturation at earlier stages than the MIH due to the GTH being able to induce OMC as well as MIH production. As mentioned, the extent of FMC acquisition is difficult to assess using an uncharacterized crude preparation like SPH; however, the ability of some of the follicles from each of the selected fish to complete oocyte maturation in vitro in response to SPH suggests some level of FMC. Nevertheless, induction of GVBD in some oocytes in response to SPH indicates that the SPH treatment was able to induce MIH production, and the objective of the present study was to identify targets of MIH and SPH at the onset of OMC.

### 3.2. Overlap of Differentially Expressed Genes Among Treatments

Despite GTHs increasing progestin production as part of their role in regulating OFM, under the study conditions, the transcriptomic response to MIH was greater than that to SPH (Figure 1B, Table S5) due in part to the dosages of the hormones used in the study, duration of exposure, and developmental stage of the follicle complexes. Many of the overlapping DEGs for S_C and M_C shared direction (68%), which is consistent with them being 17,20βP-sensitive genes. There were also 48 DEGs that overlapped the S_C and M_C comparisons that were inversely expressed (Table S7). Opposing actions may reflect changes in processes taking place in the follicle as it transitions from GTH induction of OMC to those regulated by the 17,20βP that is then produced. Among genes inversely regulated by the two hormone treatments were *follicle-stimulating hormone receptor* (100135990) and *estrogen receptor alpha 2* (100136309), receptors for two of the primary hormones decreasing in the blood at this stage of development; although Fsh abruptly increases post-ovulation [20,49]. Both genes were reduced by SPH and increased by 17,20βP. Increased follicle expression of *fshr* mRNA is associated with increased FMC in rainbow trout, and expression increases throughout GVM [14]. Increased expression of *fshr* by 17,20βP supports MIH contributing to the acquisition of FMC, although the function of increased Fshr is unclear [14,15,50]. Expression of *erα2* also increases in the ovary late in the reproductive cycle of female rainbow trout, but the narrow peak in the gene’s expression takes place at the time of peak plasma E2 concentrations, which precedes late-GVM and is therefore before 17,20βP is significantly elevated [11,45,51,52]. In addition, the expression of *erα* is higher in high FMC follicles than at maturation [15]. As previously mentioned, E2 is generally antagonistic to GTH induction of oocyte maturation in rainbow trout in part by reducing 17,20βP synthesis [9,10,11], and therefore, a reduction in estrogen receptor expression would favor OFM. Stimulation of *erα2* expression by 17,20βP would seem counter to known E2 actions in OFM.

Genes belonging to several growth factor families found to participate in OFM and ovulation [16,52,53,54,55] were inversely regulated by SPH and 17,20βP. Responsiveness of the TGFβ system to its ligands was disparately regulated with SPH reducing and 17,20βP increasing expression of *transforming growth factor beta receptor type 3-like* (110538464), a coreceptor for many TGFs. On the other hand, SPH decreased, and 17,20bP increased expression of two TGFβ system inhibitors, *follistatin-A-like* (110537003) and *breast cancer anti-estrogen resistance protein 3-like* (110521551). Expression of *epidermal growth factor receptor kinase substrate 8-like protein 1* (110491569), *insulin-like growth factor 2 mRNA-binding protein 1* (110538100), *tumor necrosis factor-inducible gene 6 protein-like* (110520259), and *tumor necrosis factor super family member 21-like* (110527836) were increased by SPH and decreased by 17,20βP. Included among the more dissimilarly expressed genes were ones generally involved with neuronal function, including *Complexin 3* (110506705), *neuromodulin-like,* (110507546), and *neurosecretory protein VGF-like* (110500364), which were all upregulated in response to SPH and downregulated in response to 17,20βP. The abundance of several genes involved with neurodevelopment in unfertilized eggs has been identified as a marker of egg quality in perch pike (*Sander lucioperca*) [56]. However, since transcription is silent in the oocyte during GVM, it is unlikely that GTH or 17,20βP treatment affects the levels of these maternal transcripts.

At the time the follicles were removed for culture, the follicles were being exposed to systemic GTHs, which in turn were stimulating steroid production, including the MIH. Therefore, gene expression in the fresh tissue compared to expression in control follicles sampled 24 h after removal from systemic regulation should reflect regulation by these hormones and other systemic regulatory factors. Indeed, patterns of expression of many genes in fresh tissues compared with control tissues overlapped with that of 17,20βP and SPH treatment compared with control tissues. In addition to reflecting the transcriptomic response to lost hormonal regulation, the comparison between fresh and incubated tissues provides insight into perturbations caused by the artificial environment of culture. The F_C comparison shared 676 DEGs with M_C or S_C treatment, and the expression of genes in F_C largely matched the direction of the hormone treatments. Shared DEGs with similar responses may reflect the removal of the follicle from systemic regulation; however, there were 85 genes in F_C, which were regulated in the opposite direction to both M_C and S_C, and 1317 DEGs were specific to F_C. Inversely expressed or F_C-specific DEGS might derive from responses to systemic or paracrine regulators other than 17,20βP or pituitary hormones present in the SPH or to culture perturbations.

### 3.3. Gene Ontology Functional Analysis

The results from GSEA analyses reflect that far more DEGs were identified in response to 17,20βP than to the SPH treatment, and many of the DEGs for SPH were similarly regulated for 17,20βP. Since all enriched GO terms and DEGs within GO terms for S_C were also found in M_C, GO term analysis of the present data provides limited insight into transcriptome responses specific to the pituitary hormones within SPH that are not transmitted through or shared with 17,20βP. The hormone treatments largely led to an enrichment of GO terms for *biological process* associated with the regulation of biological and chemical processes and response to assorted chemical and endogenous stimuli and enrichment of GO terms for *molecular function* associated with binding and growth factor activity. All enriched GO terms for *molecular function* for S-C were also enriched in M_C, and only three genes represented among the enriched GO terms for *biological process* for S-C were not found within enriched GO terms for M_C, although they may have been listed within different GO terms for the two comparisons. The only enriched GO term for S_C with unique DEGs was *negative regulation of gene expression* (GO:0010629). More DEGs and enriched GO terms might be expected for both the M_C and S_C comparisons if the tissues were sampled after a longer incubation period, allowing for more downstream effects considering that GVBD is not reached by 24 h of treatment.

A more diverse set of enriched GO terms for the *biological process* was revealed for the F_C comparison than either hormone treatment, although there was still considerable overlap. Many enriched terms specific to F_C, including *immune response*, *apoptotic process*, *response to stress,* and *defense response,* may indicate that the tissues were compromised by culture. Nevertheless, ovulation shares many events with an inflammatory response in vertebrates, including fish [40,57,58,59], and therefore, removal of the follicles from regulatory signals promoting or inhibiting progress towards ovulation would affect an overlapping subset of genes. However, if the endogenous hormonal milieu is promoting ovulatory processes, removal from the fish should not increase associated inflammatory responses. Enriched Go terms for *molecular function* for F_C are comparable to those for the hormone treatments, consistent with removal of follicles from GTH and 17,20βP stimulation contributing to many of the DEGs between fresh tissue and 24 h in culture. Investigating the effects of culture or removal of follicles from endogenous endocrine regulation was not a priority of the present study.

### 3.4. KEGG Pathway Analysis

Hormones are signaling molecules that support focusing on KEGG pathway analysis to better understand the effects of the hormone treatments. As with GO term analysis, most KEGG pathways enriched with SPH were also enriched for 17,20βP with more representative genes. Furthermore, many of the DEGs in pathways only enriched with SPH were nonetheless DEGs within other enriched KEGG pathways for the M_C comparison or DEGs for the M_C comparison but not among the enriched pathways. There were 137 DEGs among the pathways identified as enriched for S_C, of which 86 were also differentially expressed for the M_C comparison. All but 4 of the shared DEGS also shared direction, whereas 51 were only significant DEGs for S_C. The 51 DEGs specific to the S_C comparison are genes that may participate in actions by hormones in SPH not derived from or shared with 17,20βP production.

#### 3.4.1. KEGG Pathway Enrichment for the S_C Comparison

The majority of the SPH-specific DEGs belonged to pathways not also identified for 17,20βP in our analysis: *metabolic pathways* (25 DEGs), *GnRH signaling pathway* (7), *gap junction* (7), and *retinol metabolism* (6), but several were also found in *regulation of actin cytoskeleton* (9) and *insulin signaling pathway* (5) (Table S7). *Epidermal growth factor receptor-like* (110508550) was downregulated and part of four of the pathways for the S_C comparison. Epidermal growth factor (EGF) regulates both OFM and ovulation in fish [60,61,62]. *Metabolic pathways* included 79 DEGs, with 47 only found in this pathway. Among the 15 DEGs that only responded to SPH and were only found in *metabolic pathways* were three genes involved in cholesterol biosynthesis, *farnesyl-diphosphate farnesyltransferase 1* (110522339), *lanosterol 14-alpha demethylase* (110488242), and *3-hydroxy-3-methylglutaryl-CoA synthase 1* (110523168). These enzymes expressed in follicle cells are also involved in the biosynthesis of follicular fluid meiosis-activating sterol (FF-MAS) in mammalian follicles in response to GTHs and GDF9 [63,64,65]. In mammalian antral follicles, FF-MAS acts through increasing expression of the MAPK pathway enzymes to promote the resumption of meiosis [66]. Involvement of FF-MAS in fish oocyte maturation has not been established.

Among 21 DEGs for SPH for *regulation of actin cytoskeleton*, 12 were also DEGs for 17,20βP and expressed in the same direction. Several of the SPH-specific DEGs in this pathway are involved in the regulation of small GTPases. Small GTPases participate in the regulation of cytoskeletal rearrangement during oocyte maturation [67] and can participate in the response to EGF receptor activation [68]. As already mentioned, *epidermal growth factor receptor-like*, also a DEG in this pathway, is downregulated in response to SPH. Small GTPases are activated by guanine nucleotide exchange factors (GEFs). Two paralogs of the GEF *rho guanine nucleotide exchange factor 4* (110489553, 110535467) were downregulated by SPH, together suggesting SPH decreases EGF signaling. A third GEF, *son of sevenless homolog 1* (110493792), was upregulated by both SPH and 17,20βP. Furthermore, two paralogs of *brain-specific angiogenesis inhibitor 1-associated protein 2-like* (110536732, 110508650), also known as insulin receptor substrates of 53 kDA (IRSp53), were upregulated by SPH. IRSp53 controls the localization and activity of the small GTPase RAB35 [69]. Although *epidermal growth factor receptor-like* was differentially regulated in response to SPH, expression of *fibroblast growth factor receptor 4* and multiple fibroblast growth factor (Fgf) ligand genes were among DEGs modulated in this pathway in response to 17,20βP treatment (Table S8).

Integrins are heterodimeric transmembrane cell adhesion molecules with many functions, including roles in inflammation and angiogenesis [70]. Integrins are comprised of alpha and beta subunits. Treatment with SPH downregulated the expression of two alpha subunits, *integrin alpha 3b and 6b* (1104915470, 110520109). The expression of two beta subunits in this pathway was regulated by both SPH and 17,20βP. Expression of *integrin beta-5* (110501818) was decreased, whereas *integrin beta-4* (110492816) was increased in response to both SPH and 17,20βP. Treatment with 17,20βP identified five alpha and six beta integrin genes regulated in this pathway (Table S8).

Formation or maintenance of gap junctions is an important component of OMC and ovulation in fish. Increases in homologous and heterologous gap-junction coupling have been associated with GTH-induced acquisition of OMC in salmonids [15,55,71,72] and ovulation in medaka [73]. There were 11 DEGs identified in the *gap junction* pathway in response to SPH, with six sharing direction with 17,20βP treatment. An increase in *gap junction alpha-1 protein* (110529574), also known as *connexin 43* (*cx43.2*), was a shared response. Connexin 43 is a primary gap junction protein associated with increased OMC in fishes, and *cx43.2* mRNA abundance increases with OMC acquisition both in vivo and in response to GTH treatment in salmonids [15,46,72]. Connexin 43 may interact with microtubules comprised of alpha and beta tubulins to increase the stability of the microtubules [74]. Three tubulin beta chain genes were upregulated by SPH: *tubulin beta chain* (110496215), *tubulin beta-4B chain* (110488724), and *tubulin beta 2b (or beta 1)* (110525484). Steroid-mediated oocyte maturation in fish requires activation of inhibitory G-proteins [75,76,77]. The gene *guanine nucleotide-binding protein G(i) subunit alpha-2 (gnai2b)* (110494677) was upregulated by SPH but not 17,20βP. Although pertussis toxin-sensitive inhibitory G-proteins act as part of MIH transcription-independent signaling in the oocytes [76,77], PTX-sensitive G-proteins also regulate the distribution of connexin 43 within cells [78].

Despite SPH and 17,20βP increasing expression of *cx43.2*, the hormone treatments also led to changes in gene expression that would lead to inhibition of gap junction channels. Receptors for multiple growth factors that promote gap junctions were downregulated, including *epidermal growth factor receptor-like* by SPH alone, and *platelet-derived growth factor receptor beta* (110534178) and *melanoma receptor tyrosine-protein kinase* (110489727) by SPH and 17,20βP. In addition, the serotonin receptor, *5-hydroxytryptamine receptor 2A-like* (110534160), which also promotes gap junction intercellular coupling [79], was downregulated by both SPH and 17,20βP. This transcript had the greatest log2 fold change among the DEGs in this pathway, but the overall transcript number was somewhat low. Although gap junction formation and functioning have widely been shown to contribute to OMC, heterologous gap junctions were also found to maintain meiotic arrest and allow hydration during oocyte maturation in *Fundulus heteroclitus* [80,81].

A steroidogenic shift from E2 to progestin production is a critical step in OMC that is regulated by Lh [17,20,82]. Therefore, the pathway *steroid hormone biosynthesis* being an enriched pathway for SPH and not 17,20βP, the product of this shift in salmonids, can be expected. Six genes encoding four steroidogenic enzymes were identified in this pathway, including *aromatase-like* (110506809, 110506810), *estradiol 17-beta-dehydrogenase 1* (110538199), and *estradiol 17-beta-dehydrogenase 1-like* (110487538), which were downregulated, and *hydroxysteroid (20-beta) dehydrogenase 2(hsd20b2*) (110533703) was upregulated. The changes in gene expression among these enzymes are consistent with a steroid shift from E2 to the MIH during OMC [52,83]. Less expected was 17,20βP treatment having similar effects on these genes and a much stronger effect on *hydroxysteroid (20-beta) dehydrogenase 2* (8.8 vs 6.6 log2 fold change), suggesting that not only GTH but also the progestin product promotes the steroidal shift from E2 to the MIH during OFM. The enzyme *UDP-glucuronosyltransferase* (110537351), responsible for the glucuronidation of E2 and T in salmonids [84], was the only significantly differentially expressed gene in this pathway specific to SPH, and it was downregulated.

In addition to steroidogenesis, lipid metabolism has many important functions in folliculogenesis and oocyte maturation (see review [85]). *UDP-glucuronosyltransferase* was also included within the *retinol pathway* and was the only DEG in the pathway downregulated by SPH. All DEGs in this pathway were not affected by 17,20βP. Increased activity in this pathway suggests GTHs promote the metabolism of β-carotene to retinal and its metabolites, retinoic acid and retinyl esters. Upregulated were tandem copies of *bco1, beta, beta-carotene 15,15′-dioxygenase* (110526385, 110526946), an enzyme that cleaves beta carotene to retinal; *retinal dehydrogenase 1* (110535799), which catalyzes the conversion of retinal to retinoic acid; and *diacylglycerol O-acyltransferase 1* (110520733, 110506952), which converts retinol to retinyl esters. The DEGs for *diacylglycerol O-acyltransferase 1* are shared with the *glycerolipid pathway* as it also promotes triacylglycerol synthesis. Triglycerols are important for oocyte developmental competence [86]. The pathways *glycerolipid metabolism* and *glycerophospholipid metabolism* each had three DEGs specific for SPH. In addition to increased *diacylglycerol O-acyltransferase 1,* which catalyzes the final step in the synthesis of triacylglycerols, SPH decreased expression of *lipoprotein lipase* (110524209), which cleaves triacylglycerols promoting an increase in triglycerols. Both *dihydroxyacetone phosphate acyltransferase* (110490711), which is an enzyme that catalyzes the first step in ether-linked glycerolipid biosynthesis, and *phospholipase D1* (110497071), which is an enzyme that catalyzes the hydrolysis of phosphatidylcholine to phosphatidic acid, are increased.

Although an intraovarian gonadotropin-releasing hormone (GnRH)-GTH system has been identified in fish [87], the GTH subunits were not among the genes differentially regulated by SPH or 17,20βP in the present study. Together with six of the 11 DEGs associated with the *GnRH signaling pathway*, which are also DEGs associated with other enriched pathways for S_C, this does not provide strong support for SPH activating the GnRH signaling pathway.

#### 3.4.2. KEGG Pathway Enrichment for the M_C Comparison

Treatment with 17,20βP altered an array of pathways, including assorted signaling pathways, pathways involved in changes to the cell cytoskeleton, cell-to-cell contacts, and contacts between the cells of the follicle or oocyte and the extracellular matrix, and apoptosis. Many DEGs for the M_C comparison were not included among the significantly enriched pathways, further supporting 17,20βP as a pervasive regulator of processes involved in follicle development during OFM and ovulation in rainbow trout. Among the most enriched pathways identified by KEGG analysis were signal transduction pathways, including the MAPK, TGFβ, FoxO, and Wnt signaling pathways, and therefore they are discussed in detail here. The pathways in KEGG include the ligands, receptors, enzymes, transcription factors, co-regulators, inhibitors, and substrates that participate in transmitting an extracellular signal into an intracellular response. Nevertheless, assessing the impacts of changes in transcript abundance measured within the follicle complex on pathway activity is limited due to the multiple levels of regulation of signaling molecules following transcription, including translational controls, transcript and protein stability, enzyme activation, and cell types in which distinct pathways may be localized.

##### MAPK Pathway

The MAPK pathway includes multiple MAP kinases that are activated within protein cascades to convert an extracellular signal, in this case, a hormone, to a wide range of cellular responses and is active in regulating OFM and ovulation (see reviews [88,89,90]). The MAPK pathway gene expression profile represents the interacting influences of multiple pathways downstream of 17,20βP receptor activation and was the most enriched pathway for M_C with the largest number of DEGs; 124 of the 706 represented among the enriched pathways (Figure 3, Table S8). When these genes were themselves used for KEGG pathway analysis, many of the pathways identified as enriched in the original analysis were again identified as enriched, demonstrating the widespread impact of the MAPK signaling pathway on 17,20βP actions in the post-vitellogenic follicle complex. Indicative of the composite nature of the MAPK pathway and the effects of 17,20βP on gene expression, eight different dual specificity phosphatases (DUSPs) (*dusp1, 2, 4, 5, 6, 7, 8 and 8a*) were differentially expressed. DUSPs are largely transcriptionally regulated and play a pivotal role in regulating kinase activation, and they are, in turn, responsive to MAPK levels (Jeffrey 07 [91]). DUSPs show specificity for MAPK substrates, which they downregulate, and members of all three subfamilies of DUSP, nuclear and cytosolic, were upregulated in response to 17,20βP.

Many tyrosine kinase receptors (RTK) and their ligands were differentially expressed in response to 17,20βP, again demonstrating the converging influences on activation of MAPK pathways within the follicle in response to the progestin. Platelet-derived growth factors (PDGF), vascular endothelial growth factors (VEFG), placental growth factors (PLGF), and their receptors are part of the PDGF/VEGF family. PDGFs and VEGFs are similar in structure, and all are dimers of disulfide-linked polypeptide chains, with each chain encoded by a different gene [92]. Expression of PLGF and multiple VEGF ligands was decreased in response to 17,20βP, but not PDGF ligand genes. Receptors for PDGF (PDGFR) and VEGF (VEGFR) are also formed by alpha and beta subunits, encoded by separate genes, that dimerize upon binding to the ligand. Receptor specificity is not well characterized, especially in fish. Treatment with 17,20βP decreased *vascular endothelial growth factor receptor kdr-like* (100136647) and *platelet-derived growth factor receptor alpha* (110523003, 110521671) and increased two PDGFRB transcripts: *platelet-derived growth factor receptor beta* (110488623, 110504831). But, as mentioned earlier, both 17,20βP and SPH treatment decreased a third copy of *platelet-derived growth factor receptor beta* (110534178). In addition, expression of *RAC-alpha serine/threonine protein kinase* (110497971) (aka. AKT1, PKB) and *RAC-beta serine/threonine protein kinase* (110507997) (aka AKT2), which are activated by many growth factors including PDGF and VEGF, were increased in response to 17,20βP.

Receptors for FGF, EGF(ErbB), insulin (INS), and ephrin (Eph) family peptides are also RTKs. Fibroblast growth factors were among the most highly upregulated and downregulated growth factors within the MAPK pathway. Nevertheless, *fibroblast growth factor 9* (110505284) was the most highly upregulated gene in the MAPK pathway and had over 10 log2 fold more transcripts than the other genes. Furthermore, *fibroblast growth factor receptor 4* was also highly expressed and upregulated, supporting 17,20βP increased FGF signaling in the follicle. Involvement of receptors EGFR/ErbB in fish OFM has been demonstrated [62,93]; however, *epidermal growth factor receptor-like* was downregulated by SPH, but not 17,20βP, and *ErbB signaling pathway* was not a highly enriched pathway in the M_C comparison. The receptor, *receptor tyrosine-protein kinase erbB-3* (110491976) was upregulated, but this ERB3 binds heuregulin and neuregulin-2 [94,95]. Still, the *ErbB signaling pathway* was found to be highly enriched when *MAPK pathway* DEGs were analyzed independently. Among DEGs for other RTKs in the *MAPK pathway*, mRNAs for *melanoma receptor tyrosine-protein kinase* were decreased, and *insulin receptor* (100135871) increased. In addition, *ephrin-A2* (10486932) was upregulated, whereas *ephrin-A5* (110509759) and *ephrin-A5b* (110523550) were downregulated. In total, six classes of growth factor RTKs belonging to RTK classes I–V and IX, or their ligands, were among DEGs modulated within the MAPK kinase pathway in response to 17,20βP treatment.

##### FoxO Pathway

The FoxO signaling pathway leads to signaling by the FoxO system of transcription factors (see reviews [96,97]). The FoxO transcription factors *forkhead box protein O3* (110529912) and *forkhead box O3b(foxo3b)* (110522360) were among the most highly upregulated genes in the pathway, and *forkhead box protein O1-A* (110534227) was also upregulated. FoxO proteins were found to be upregulated during the resumption of meiosis in rainbow trout [40]. Direct promoters of FoxO, including *P38alpha mitogen-activated protein kinase 14A* (110494762) and *cyclin-dependent kinase 2* (110528231), were also upregulated in response to 17,20βP treatment. In contrast, a positive modulator of FoxO signaling, *Stat3* (100136756, 110538194), was among the most highly expressed transcripts that were downregulated in this pathway. A number of growth factors and interleukin 6 regulate Stat3, and Fsh, Lh, and E2 have also been shown to increase the il-6/stat3 pathway in ovarian surface epithelial cells [97]. Expression of interleukin 6 (interferon, beta 2) (il6) (100136689) was downregulated in response to 17,20βP. In addition, many genes along the insulin/IGF pathway that inhibit FoxO action [98] were also upregulated, including key direct regulators and inhibitors of FoxO3 and FoxO1: *RAC-alpha serine/threonine protein kinase*, and *RAC-beta serine/threonine protein kinase,* as already mentioned. Steroid-induced oocyte maturation is dependent on the activation of the phosphatidylinositol 3-kinase/akt signal transduction pathway in many vertebrates [99,100]. Expression of the serine/threonine protein kinases (SGK), *serine/threonine protein kinase Sgk1* (110488097), and *serine/threonine protein kinase Sgk2* (110492337) were also upregulated with *serine/threonine protein kinase Sgk1* being the most abundant transcript in the pathway and among the most highly upregulated. Serine/threonine protein kinase genes have a progesterone response element but also many transcription binding sites [101,102], making it difficult to determine the ligand or ligands contributing to their increased expression. The SGK, Sgk1, is required for the resumption of meiosis in a wide range of animals, including starfish [103] and mouse [104], but its involvement in oocyte maturation in fish has not been established. However, an increase in *sgk2* expression during follicle maturation in fish, including rainbow trout, has been reported [40].

Treatment with 17,20βP led to the activation of many FoxO target genes with diverse actions including genes involved in metabolism (*glucose-6-phosphatase*, 100135885, 110499065), immuno-regulation (*Krueppel-like factor 2*, 110501271), apoptosis (*BCL2/adenovirus E1B 19 kDa protein-interacting protein 3*, 110531588), but mostly cell cycle regulation (*cyclin B3*, 110531416; *cyclin-G2*, 110536901; *cyclin-dependent kinase inhibitor 1B*, 110500225; *polo-like kinase 2b (Drosophila)*, 110536158; *serine/threonine protein kinase PLK2* (110525781); and several *gadd45* mRNAs). The DEG *polo-like kinase 2b (Drosophila)* was one of the most upregulated and highly expressed DEGs, second in counts to *serine/threonine protein kinase Sgk1*, and specific to the FoxO pathway. Polo-like kinases (PLK) participate in the resumption of meiosis in mammals [105,106], and *plk1*, *plk2*, and *sgk-like* increase in the ovarian follicle as they approach oocyte maturation in spotted scat (*Scatophagus argus*), supporting similar actions in fish [35]. Treatment with 17,20βP increased the abundance of seven *gadd45* transcripts, including alpha, beta, and gamma forms, with both *gadd45 alpha* and *beta* forms being highly expressed. Bobe et al. 2006 [40] also reported upregulation of *gadd45* at the time of resumption of meiosis in this species. The primary action of GADD45 is to inhibit cell-cycle progression in response to stress, but it also serves as a marker of follicle apoptosis in mammals [107]. Luteinizing hormone induction of ovulation in fishes acts via a TNFa-dependent increase in prostaglandin F2a [16]. Many DEGs in the FoxO signaling pathway in the present study also responded to salmon Lh treatment of preovulatory follicles of brown trout *Salmo trutta* [16]. Expression of the pro-apoptotic gene *TNF superfamily member 10-like protein* was decreased in response to Lh in brown trout and both 17,20βP and SPH in rainbow trout (*TNF superfamily member 10-like protein,* 100136317). As in the present study using 17,20βP, Lh also increased mRNA transcripts for *gadd45*, *forkhead box 03a*, and *polo-like kinase 2* in brown trout. Members of the TGFβ system and enriched DEGs for the *TGFβ signaling pathway*, including *smads* and *tgfb* ligands, were also included within the *FoxO signaling pathway* as DEGs modulated by 17,20βP treatment.

##### TGFβ Pathway

The TGFβ superfamily includes the TGFβ, bone morphogenetic protein (BMP), and activin (Act) families, and growth and differentiating factor 9 (GDF9) (see review [108]). Members of this superfamily participate in regulating nearly all major developmental processes in ovarian follicle development (see review [109]), including OFM and ovulation in rainbow trout and other fishes (e.g., [15,45,53,54,110,111,112,113,114,115]). Treatment with 17,20βP had widespread effects on TGFβ/BMP/activin system gene expression. TGFβ1 inhibits GTH and MIH-induced oocyte maturation in zebrafish [54,112], although little change in expression is seen during the preovulatory period in rainbow trout [15]. TGFβ ligands *transforming growth factor, beta 1a* (101268942), *transforming growth factor, beta 2* (110519367), and *transforming growth factor beta-2 proprotein* (110490140) were upregulated whereas the level of mRNA for *transforming growth factor beta-3 proprotein* (110505545) was reduced and very low. TGFβ is usually found in a latent form complexed with a latency-associated peptide (LAP) and associated with a latent transforming growth factor beta-binding protein (LTBP). The complex is tethered to the cell surface by transforming growth factor activator LRRC32 [116,117]. The LTBP, LTBP-3, has been shown to be upregulated by E2 acting through erα in rainbow trout [118]. Here, we find increased expression of *transforming growth factor beta activator LRRC32* (110537705), which maintains TGFβ in a latent state during storage in response to 17,20βP. In addition, *thrombospondin-1* (110497949), a major activator of TGFβ1 [119], was the most highly expressed gene in the study, and expression was increased by 17,20βP, and even more so with SPH. Expression of *thrombospondin 1b* (110522588) was also highly upregulated by 17,20βP but did not respond to SPH. Thrombospondin-1 (THBS1) also has pro-angiogenesis and anti-angiogenic properties in the developing follicle and corpora lutea in mammals and is critical for ovulation and supports the resumption of meiosis in primates [120,121].

Treatment with 17,20βP affected the expression of five BMP ligands. The BMP *bone morphogenetic protein 6* (110489762) was upregulated and highly expressed, whereas *bone morphogenetic protein 16* (110507419), a novel BMP2/4 relative in fishes and reptiles [122], was downregulated. Other BMPs were upregulated but expressed at very low levels, including *bone morphogenetic protein 4* (110505700), *bone morphogenetic protein 7* (110517304), and *bone morphogenetic protein 2-like* (110491444). Another copy of *bone morphogenetic protein 2-like* (110503302) was more highly expressed but only enriched in response to SPH treatment (Table S7). The receptor mRNAs, *bone morphogenetic protein receptor type-1B* (110536815) and *bone morphogenetic protein receptor type IBb* (110522929), and the BMP co-receptor *repulsive guidance molecule A* (110524278) were highly expressed and upregulated. Despite the expression of *bone morphogenetic protein 4* and *bone morphogenetic protein 7* being very low in the present study, both were found to be significantly increased in maturation stage follicles in rainbow trout [15]. BMP6 regulates iron homeostasis by increasing hepcidin expression [123], a peptide hormone that inhibits the movement of iron into the circulatory system by inhibiting the iron export protein ferroportin in the liver. Expression of *hepcidin-like* (100135935) was downregulated in the follicle complex in the present study. Additionally, three transferrin receptor-1 transcripts were in the *TGFβ signaling pathway* modulated in response to 17,20βP. Transferrin is a glycoprotein that transports iron in the blood and releases the iron into the cell by receptor-mediated endocytosis upon binding to the TRF1 receptor. Two of the transcripts were downregulated (*transferrin receptor 1b*, 11053536; *transferrin receptor protein 1*, 100653457), but the most highly expressed was upregulated (*transferrin receptor 1a*, 110509090). The *Ferroptosis pathway* was a KEGG pathway enriched by 17,20βP treatment (Figure 3), but it is unclear if BMP6 is involved in ferroptosis in the follicle complex, and *bone morphogenetic protein 6* was not included as a DEG in this enriched pathway.

Activin system regulation of OFM has received much attention, including in rainbow trout. Activin has been shown to stimulate oocyte maturation in fish, and the activin system has been well described in the zebrafish follicle [53,60,61,111]. Activins and inhibins are protein complexes that share dimer subunits, making it difficult to determine which of the growth factors are increased based on changes in the expression of the sub-unit mRNAs. Bobe et al., 2004 [15] concluded that increased activin-βA (*inhba*) was associated with the acquisition of FMC and inhibin (*inhba* and *inha*) with oocyte maturation in rainbow trout [15]. In general, the activin system was inhibited in response to 17,20βP in the present study. Only inhibin beta chains were differentially expressed and were highly downregulated (*inhibin subunit beta Ab*, 110509121; *inhibin beta A chain*, 110535572; *inhibin beta B chain,* 110520286, 110501797), with *inhibin subunit beta Aa* (110490008) being most highly expressed and downregulated. In a previous study, expression of *inhba,* but not *inhbb* or *inha*, was strongly downregulated in response to 17,20βP in vitro in rainbow trout follicles [110]. The reason for the difference in response of *inhbb* transcripts to 17,20βP in our two studies is unclear. The response to 17,20βP was strong, showing greater than a 5 log2 fold change reduction in the *inhibin beta B chain* (110501797), and a similar reduction was found with SPH. As mentioned, homodimers or heterodimers of the disulfide-linked beta subunits derived from these changes in expression can represent a reduction in multiple activin or inhibin proteins. Furthermore, an activin type 2 receptor was also decreased (*activin A receptor type 2Aa*, 110520186), and expression of the major inhibitor of the activin system, follistatin (*follistatin*, 100301646; *follistatin-A*, 110537003), was increased, further supporting a decrease in activin activity in the follicle complex in response to 17,20βP.

The TGFβ superfamily signals through the activation of SMAD proteins, which include receptor SMADs, co-SMADs, and inhibitory SMADs. The receptor-smad, *smad1* (*mothers against decapentaplegic homolog 1*, 110497366, 110534530) of the BMP signaling pathway was the only receptor-regulated smad differentially expressed, and it was upregulated. The co-smad, *smad4* (*mothers against decapentaplegic homolog 4*, 101268921, 110488280, 110503878) was downregulated whereas the inhibitory SMADs, *smad6* and *smad7* were disparately regulated, with *smad6* (*SMAD family member 6b*, 110509922, 110510499) downregulated and *smad7* (*SMAD family member 7*, 110536948) upregulated. Increased *smad1* and *BMPR1b* transcripts, as mentioned earlier, support increased sensitivity to BMPs in response to 17,20βP. On the other hand, *homeobox protein TGIF1* (110528482, 110493969), a co-repressor of smad2 [124], a receptor-smad for activin and TGFβ signaling, was upregulated. In addition, *zinc finger FYVE domain-containing protein 16* (110523341), which promotes the receptor Smad–Smad4 complex formation, was downregulated [125].

The TGFβ/BMP system is also controlled by inhibitors at all levels of regulation in addition to the inhibitory smads and follistatin already mentioned. Many inhibitors were differentially regulated in response to 17,20βP. Among the downregulated inhibitors were the transcription factor ID1 (*DNA-binding protein inhibitor ID-1*, 110527919, 110532286, 100136776), an inhibitor of DNA binding [126], and *gremlin 1b* (110497956), a secreted protein BMP antagonist [127]. Noggin is also a secreted polypeptide that binds and inhibits most BMPs, and three Noggins are found in fish [128]. Expression of *noggin-2-like* (110485721) was highly expressed and downregulated, whereas *noggin 3* (110502770) was upregulated but expressed at very low levels. *Ski* (*ski-like protein* (110509188, 110530777) and *bambi* (BMP and activin membrane-bound inhibitor (Xenopus laevis) homolog a, 100301651) were also upregulated in response to 17,20βP, with *bambi* being highly expressed and the most upregulated of the inhibitors. Ski is a transcriptional co-repressor of smad proteins [129], and BAMBI is a type I pseudoreceptor that lacks an intracellular serine/threonine kinase domain required for signaling [130]. BAMBI can interact with and inactivate most type-II receptors. We have previously shown *bambi* to be increased in OMC follicles and highly upregulated in rainbow trout follicles in response to 17,20βP [45,110]. BAMBI can also promote Wnt/β-catenin signaling [131].

##### Wnt Pathway

The Wnt signaling pathway includes canonical and noncanonical pathways mainly mediated by Wnt proteins. The canonical or Wnt/β-catenin pathway is mainly mediated by Wnt proteins and consists of the extracellular signal, a membrane, a cytoplasmic, and a nuclear segment (see review [132]). Both the canonical and noncanonical pathways participate in the regulation of ovarian follicle development (see review [133]). Treatment with 17,20βP primarily modulated genes of the canonical Wnt pathway. Wnt ligands bind to the transmembrane receptors Frizzled (Fzd) and low-density lipoprotein receptor-related proteins 5 and 6 (LRP5/6) to activate the Wnt canonical pathway. Binding of Wnt inhibits the degradation of the signaling molecule β-catenin. In the absence of Wnt binding, β-catenin is phosphorylated and ubiquitinated, leading to proteosome-mediated degradation by the β-catenin destruction complex [134]. The destruction complex includes glycogen synthase kinase 3 (GSK-3), casein kinase 1 (CK1), Axin, adenomatous polyposis coli (APC) protein, and β-TrCP. When Wnt binds to its receptor, it recruits Dishevelled (Dvl), which causes the disassembly of the complex. β-Catenin then accumulates and translocates to the nucleus, where it interacts with the transcription factors of the lymphoid enhancer-binding factor/T-cell factor (TCF/LEF) family to activate the transcription of Wnt target genes.

The Wnt system in fish has been investigated primarily for its role in sex determination and early gametogenesis, including in rainbow trout [135,136]. Single-cell transcriptome analysis of the zebrafish ovary identified expression of *wnt4* and *wnt9b* in follicle cells, *wnt9a* in stromal cells, *wnt11* in stromal and theca cells, and *wnt8a* in the oocyte demonstrating Wnt system activity throughout the follicle complex [137]. In catfish (*Clarius batrachus*), *wnt4* expression is elevated prespawning, whereas *wnt5* is elevated during the spawning period in the follicle cells and is increased in response to hCG injection [138]. The Wnt signaling pathway was generally upregulated in response to treatment with 17,20βP in the present study, supporting a role in OFM in fish. The Wnt proteins *wingless-type MMTV integration site family, member 2Ba* (110527771), and *wingless-type MMTV integration site family, member 9A* (110530487), the most highly expressed Wnt ligand, were highly upregulated in response to 17,20βP, whereas *wingless-type MMTV integration site family member 4a1* (101268970) and *wingless-type MMTV integration site family, member 5a* (110492557) were downregulated but expressed at lower levels.

Transcripts for Frizzled 1 (*frizzled-1-like*, 110511951), 7 (*frizzled-7-A*, 110495872, 110527486; *frizzled class receptor 7a*, 110517403), and 8 (*frizzled class receptor 8a,* 110535536), were upregulated while Frizzled 2 (*frizzled-2*, 110485166, 110538204) were downregulated in response to 17,20βP. Expression of LRP5, *low-density lipoprotein receptor-related protein 5* (110509621), was also increased. In addition, the Wnt agonist *R-spondin-3* (110511593) and its receptor *leucine-rich repeat-containing G-protein coupled receptor 4* (110506651, 110526521), were upregulated in response to 17,20βP [139].

Downstream β-catenin accumulation and signaling were also promoted by 17,20βP treatment. Expression of *segment polarity protein disheveled homolog DVL-1* (110532465) was increased, and the *coiled-coil domain containing 88C* (110497742), a Dvl-binding protein that impedes Wnt signaling, was reduced [140]. There was increased expression of several PRICKLE (prickle planar cell polarity protein) transcripts (*prickle homolog 1b*, 110499700; *prickle homolog 3*, 110532495; *prickle-like protein 1*, 110499701, 110499974; *prickle-like protein 2*, 110527970). PRICKLE is generally part of noncanonical Wnt pathways but has also been shown to promote Dvl ubiquitination/degradation [141,142]. The steroid increased mRNA levels of one component of the destruction complex, *glycogen synthase kinase binding protein* (11048668), but decreased another, *axin-2* (110486806). The transcripts *casein kinase II subunit alpha* (110496662) (aka CK2), *cAMP-dependent protein kinase catalytic subunit beta* (110508616) (aka PRKACB), *testis catalytic subunit of cyclic adenosine 3′,5′-monophosphate dependent protein kinase* (100136597) (aka PKA), and *histone acetyltransferase p300* (110502858) were upregulated which favors Wnt signaling. An increase in CK2 is required for Wnt/ β-casein signaling [143]. The enzyme PRKACB activates PKA, which in turn stabilizes β-catenin [144], and p300 is a transcriptional co-activator of β-catenin [145]. The primary transcription factor co-regulators of β-catenin, *transcription factor 7-like 2* (110537729) and *lymphoid enhancer-binding factor 1* (110497329), were expressed at very low levels but were upregulated in response to 17,20βP. Nevertheless, inhibition was indicated based on increased expression of *leucine zipper putative tumor suppressor 2 homolog* (110491536, 110502653), a protein that interacts with and represses the transactivation of β-catenin [146].

When β-catenin binds to TCF/LEF to activate the transcription of target genes, it displaces groucho/TLE. When TCF/LEF is bound to groucho/TLE it acts as a transcriptional repressor [147]. Three members of the TLE family, members 1, 2, and 3 (*TLE family member 1, transcriptional corepressor*, 110525269; *TLE family members 2, transcriptional corepressor a*, 110524327; *TLE family member 3, transcriptional corepressor a*, 110512674) were upregulated in response to 17,20βP. Although groucho/TLE suppresses the expression of Wnt target genes, it does not block Wnt signaling. Canonical Wnt signaling can also be inhibited by a number of naturally secreted antagonists, including DKK1,2, secreted frizzle (SFRPs), and sclerostin, which is encoded by the SOST gene [148,149,150]. Expression of *dickkopf-related protein 2* (110497321), *dickkopf-related protein 2-like* (110534949), and *sclerostin* (110499072) were downregulated, which further supports 17,20βP stimulates the wnt pathway, but they were expressed at much lower levels than *secreted frizzled-related protein 3* (110520325), which was upregulated. Furthermore, as previously mentioned, *BMP and activin membrane-bound inhibitor (Xenopus laevis) homolog a* was highly expressed and upregulated in response to 17,20βP; however, whereas BAMBI acts as a receptor antagonist in the TGFβ/BMP/activin system, BAMBI positively modulates Wnt/ β-catenin [131]. The functional β-catenin-TCF4 complex β-catenin has been found to directly upregulate *Bambi* in colorectal tumor cells [151]. Other than an increase in SFRP3, 17,20βP treatment affected the expression of these secreted peptides in ways that support Wnt signaling.

## 4. Materials and Methods

### 4.1. In Vitro Incubations Bioassay and Sample Selection

The ovarian follicles used in the study were about 2-year-old rainbow trout from stocks maintained at the USDA National Center for Cool and Cold Water Aquaculture (NCCCWA, Kearneysville, WV, USA). Fish were reared indoors under an artificial ambient photoperiod in continuous-flow treated spring water at 13 ± 1 °C, with dissolved oxygen content near air saturation. Fish were fed with Zeigler Broodstock diet (Zeigler Bros. Inc., Gardners, PA, USA) at 1% body weight daily starting about six months before spawning. Fish without signs of malnourishment or significant external signs of infection or injury were euthanized with 150 mg/L of tricaine methanesulfonate (MS-222; Western Chemical, Ferndale, WA, USA) before removal of the ovaries. Harvested ovarian tissue was placed into a sterile ice-cold trout mineral medium (TMM; Bobe et al., 2003 [14]) for follicle isolation. Four samples of five large intact follicles were isolated from each ovary and immediately frozen in liquid nitrogen and served as the fresh tissue treatment. An additional ovarian fragment from each fish was cleared in Davidson’s solution (2 parts 37% formaldehyde: 3 parts 95% EtOH: 1 part acetic acid: 3 parts H_2_O) for ~15 min to identify fish with oocytes near late-GVM. Oocytes were considered late-GVM stage when the GV was between 75% of the way towards the periphery of the oocyte without reaching the periphery. Nine fish at or approaching late GVM were selected for the study, although tissues from only three fish were selected for RNA-seq analysis based on the assessment of FMC.

Large follicles from each fish were isolated under a dissecting microscope for use in the study. In total, 160 follicles were isolated from each fish. Half of the follicles from each fish were used for assessment of FMC based on GVBD response at ~96 h in incubation 1, and the other half for transcript response to incubation and hormone treatments at 24 h in incubation 2. Both parts of the study utilized an in vitro assay for follicle maturation, as described previously [11,14,110]. There were four replicate wells for each hormone treatment for each fish (designated control, MIH, and SPH) for each incubation of the study. In incubation 1, the tissues were incubated for ~96 h and then fixed in Davidson’s solution to score GVBD as a marker for the initiation of the resumption of meiosis. In incubation 2, five follicles per well were collected into liquid nitrogen for mRNA analysis after 24 h. For each replicate, 20 follicles were placed in a well of a Falcon 12-well culture plate (Becton Dickinson, Franklin Lakes, NJ, USA) with 2.7 mL culture medium (TMM) on an orbital oscillator at 80 rpm placed in an incubator at 10 °C. After 1 h pre-incubation, an additional 0.9 mL of culture medium was added to the well as control, or 0.9 mL of 1160 nM 17,20βP (Steraloids Inc., Newport, RI, USA), to obtain a final concentration of 290 nM 17,20βP, or the medium was fully replaced with 4 mL of 33 mg/mL SPH (Argent Chemical Laboratory, Redmond, WA, USA). All frozen samples were transferred to a −80 °C freezer until RNA isolation. Three fish, #1, #5, and #6, were chosen for RNA sequencing based on a high percentage of follicles completing GVBD in response to 17,20βP and some follicles completing GVBD in response to SPH.

### 4.2. RNA Isolation and RNA-Sequencing

Samples for RNA isolation from incubation 2 were composed of five follicles from each replicate well for each treatment from the three fish selected for RNA-seq analysis. Tissues were homogenized in Tri Reagent (Sigma, St. Louis, MO, USA) with an MM300 TissueLyser (Retsch Inc., Haan, Germany). Total RNA was isolated following the manufacturer’s protocol with the modification of using Phase Lock Gel tubes (5 PRIME, Inc., Gaithersburg, MD, USA), Phase Separation Reagent (Molecular Research Center, Cincinnati, OH, USA), and the inclusion of a high salt solution (Molecular Research Center, Cincinnati, OH, USA) to the precipitation step to remove excess glycosylated proteins. The isolated RNAs were then treated with DNase (Promega, Madison, WI, USA) and quantified using a NanoDrop ND-1000 (Thermo Scientific, Wilmington, DE, USA) with an average 260/280 ratio greater than 1.8–1.9. An aliquot of RNA from each well for a given treatment replicate was pooled, and about 4 μg of RNA per sample was submitted to the Roy J. Carver Biotechnology Center, University of Illinois at Urbana-Champaign, for RNA-seq analysis. The submitted RNAs were further quality-checked by the Center and used for library construction with the TruSeq RNA Library Prep kit. The RNAs for the 12 libraries were from the fresh, control, MIH, and SPH treatments for fish #1, #5, and #6. Single-end sequencing of 100 bp for each of the 12 libraries was barcoded and sequenced in a total of 6 lanes using the Illumina HiSeq 2000 platform (San Diego, CA, USA).

A total of 307,465,653 single-end RNA-seq reads were obtained from the 12 samples. STAR, which is a splicing-aware sequence aligner [152], was used to align the RNA-seq reads to the rainbow trout reference genome (GCF_002163495.1) [153]. The raw sequencing data used in the manuscript was deposited in the NCBI Sequence Read Archive database (BioProject Accession: PRJNA389564; SRA Accession: SRP108755). In building the STAR reference library, intron junction sites in the genome were marked using the NCBI RefSeq annotation data on this reference. Reads mapped to the genes were counted using htseq-count [154]. On average, 74.29% of the reads were uniquely mapped to the reference, and reads that mapped to multiple locations (16.50%) were excluded.

### 4.3. Differential Expression Analysis

The unnormalized raw count data were processed with the R packages DESeq2 [155] and edgeR [156] to test for differential expression of the genes between the samples of each pair of treatments. The treatment comparisons were for the MIH, 17,20βP, versus control (M_C), SPH versus control (S_C), and fresh tissue versus control (F_C) follicles. In each comparison, biological replicates from three samples were used for each treatment group. Differentially expressed genes were selected based on the adjusted *p*-value (padj) calculated with the Benjamini–Hochberg method [157], and those with padj < 0.05 from both DESeq2 and edgeR were selected for further analysis.

### 4.4. Gene Ontology and KEGG Pathway Analysis

The Database for Annotation, Visualization, and Integrated Discovery (DAVID) gene functional classification tool [158] was used for Gene ontology (GO) analysis and KEGG pathway analysis of the DEGs identified by both programs. Animal database Oncorhynchus mykiss, Taxonomy ID: 8022, was used for the analyses. Prior to conducting the DAVID analyses, the gene IDs of the DEGs were converted to the most recent rainbow trout reference genome (GCF_013265735.2) [159]. DAVID pathway analyses were conducted using the default parameters: ease score 0.1, kappa score 0.35, and minimum gene count of 2 with the DAVID knowledgebase v2023q2, which was released on 23 June 2023.

### 4.5. Quantitative Real-Time PCR Analysis

Quantitative real-time PCR (RT-qPCR) was carried out in tetraplicate to verify DETs through quantification of selected gene expressions. Two μg of DNase-treated RNAs were converted to cDNA for each replicate using an M-MLV reverse transcription kit following the manufacturer’s protocol (Life Technologies Corporation, Carlsbad, CA, USA), and rainbow trout elongation factor-1-alpha (ef1α) was used as reference gene. RT-qPCR was performed in quadruplicate for each of the cDNA samples using an ABI 7900HT sequence detection system (Applied Biosystems, Foster City, CA, USA). Each reaction consisted of 1.5 µL of 1:5 diluted cDNA, 0.25 to 1 µM of each primer, and 1× SYBR Green PCR Master Mix (Applied Biosystems, Foster City, CA, USA). The thermal cycling profile was 50 °C for 2 min; 95 °C for 10 min; and 40 cycles of 95 °C for 30 s, 60 °C for 20 s, and 72 °C for 30 s. A final dissociation step was performed to assess the specificity of the reaction. Relative quantification of the transcripts was estimated by the standard curve method, and Mean differences in expression levels were reported as relative fold changes using the lower expression value as a calibrator. The primers used in the RT-qPCR are listed in Supplemental Table S1. The Spearman’s rank correlation was estimated for the results of RT-qPCR and RNAseq by R (Version 4.4.1).

## 5. Conclusions

The present study identifies many genes and pathways throughout the follicle that are responsive to MIH and SPH treatment and to removal from systemic regulation. A total of 5292 were identified among the three pairs of comparisons, with the 17,20βP treatment yielding the greatest number of DEGs with 1954 upregulated and 1747 downregulated genes. Extensive overlap in transcript responses to SPH and 17,20βP treatment suggests many gonadotropin actions leading to the acquisition of maturational and ovulatory competence are mediated in part by gonadotropin induction of 17,20βP synthesis. Treatment with 17,20βP modulated a wide range of processes involved with OFM and ovulation. KEGG analysis identified a diverse set of pathways altered in response to 17,20βP treatment. Signaling pathways, including MAPK, TGFβ, FoxO, and Wnt signaling pathways, were among the most significantly enriched pathways altered by 17,20βP treatment, suggesting pervasive influences of 17,20βP on actions of other endocrine and paracrine factors in the follicle complex. The follicle is a mix of cells contributing to these changes in expression, including granulosa, theca, and stromal cells of the somatic follicle, as well as the oocyte. Ascribing components of molecular regulation revealed by the study to the regulation of specific reproductive events is beyond the ability of this study. Nevertheless, mechanisms of molecular regulation of ovarian follicle development worthy of further study to better understand hormonal regulation of OFM and ovulation that may contribute to egg quality can be identified from the present investigation.

## Figures and Tables

**Table 1:** Germinal vesicle breakdown in follicle-enclosed oocytes in response to 17,20βP, SPH, and control treatments.

	17,20βP		SPH		Control	
Fish	*n*	Germinal Vesicle Break Down (%)	*n*	Germinal Vesicle Break Down (%)	*n*	Germinal Vesicle Break Down (%)
Fish 1	80	97	80	19	78	0
Fish 2	80	46	80	0	73	0
Fish 3	80	0	70	0	75	0
Fish 4	86	49	90	0	70	0
Fish 5	91	80	94	7	73	0
Fish 6	88	100	92	8	83	0
Fish 7	92	0	92	0	62	0
Fish 8	92	100	92	97	57	0
Fish 9	92	85	92	0	79	0

The *n* value indicates the total number of follicles scored for GVBD.

**Table 2:** Messenger RNA sequencing statistics.

Sample	Raw Reads	Uniquely Mapped Reads	Multiple Mapped Reads	Unmapped Reads
Fish 1 Control	25,658,354	18,810,404	4,498,966	2,348,984
Fish 1 Fresh	36,409,248	27,646,680	5,520,008	3,242,560
Fish 1 MIH	23,046,303	16,829,377	4,015,458	2,201,468
Fish 1 SPH	24,242,785	18,596,416	3,566,571	2,079,798
Fish 5 Control	23,601,043	17,763,451	3,689,144	2,148,448
Fish 5 Fresh	23,851,239	17,702,404	3,967,678	2,181,157
Fish 5 MIH	34,136,174	25,172,087	5,599,459	3,364,628
Fish 5 SPH	25,614,409	19,756,515	3,700,793	2,157,101
Fish 6 Control	18,293,468	13,333,335	3,256,496	1,703,637
Fish 6 Fresh	24,027,278	17,554,537	4,242,701	2,230,040
Fish 6 MIH	22,423,964	16,178,584	4,097,088	2,148,292
Fish 6 SPH	26,161,388	19,384,938	4,254,896	2,521,554

**Table 3:** Significantly enriched gene ontology terms for *Biological Process* for each comparison. The count of the number of DEGs associated with the GO term, the GO term level, and *p*-values are indicated.

Biological Process			
**17,20βP versus Control (M_C)**	**Count**	***p*-Value**	**Level**
**Biological regulation**	129	0.002	1
Regulation of biological process	114	0.009	2
Negative regulation of biological process	32	0.002	3
**Cellular process**		NS	1
Cellular response to organic substance	15	0.007	4
Cell-cell signaling	14	0.005	3
Signal transduction		NS	3
Enzyme-linked receptor protein signaling pathway	10	0.006	6
Transmembrane receptor protein tyrosine kinase signaling pathway	8	0.010	7
Regulation of cellular process	100	0.009	2
Negative regulation of cellular process	28	<0.001	3
Negative regulation of cellular metabolic process	13	0.005	4
**Localization**		NS	1
Regulation of peptide secretion	4	0.007	8
Regulation of localization	17	0.002	2
Regulation of transport	17	<0.001	3
**Response to stimulus**	98	0.005	1
Response to chemical	35	<0.001	2
Response to nitrogen compound	10	0.005	3
Response to organic substance	30	<0.001	3
Response to endogenous stimulus	19	<0.001	2
Response to hormone	13	<0.001	3
Response to peptide hormone	8	0.007	4
**SPH versus Control (S_C)**	**Count**	***p*-Value**	**Level**
**Biological regulation**		NS	1
Regulation of biological process	30	0.030	2
**Metabolic process**		NS	1
Negative regulation of gene expression	6	0.027	6
**Reproductive process**		NS	1
Female gamete generation	3	0.007	4
**Response to stimulus**	28	0.008	1
Response to endogenous stimulus	4	0.043	2
**Fresh versus Control (F_C)**	**Count**	***p*-Value**	**Level**
**Biological regulation**		NS	1
Regulation of biological process	84	<0.001	2
Signaling	39	0.028	3
**Cellular process**		NS	1
Cell motility	9	<0.001	2
Cellular metabolic process		NS	2
Regulation of phosphate metabolic process	9	0.001	5
Cell proliferation	8	0.001	2
Cell death		NS	2
Apoptotic process	9	0.029	4
Regulation of cellular process	66	0.004	2
**Immune system process**		NS	1
Immune response	21	<0.001	2
Regulation of immune system process	10	0.004	2
**Localization**		NS	1
Establishment of localization		NS	2
Phagocytosis	4	0.014	6
Regulation of localization	14	<0.001	2
Regulation of transport	11	0.006	3
**Metabolic process**		NS	1
Regulation of metabolic process	33	0.004	2
Biosynthetic process		NS	2
Regulation of gene expression	27	0.008	5
**Response to stimulus**	78	<0.001	1
Response to chemical	24	<0.001	2
Response to stress	27	0.004	2
Defense response	19	<0.001	3
Inflammatory response	10	<0.001	4
Regulation of response to stimulus	19	0.003	2

NS indicates not statistically significant.

**Table 4:** Significantly enriched gene ontology terms for *Molecular Function* for each comparison. The count of the number of DEGs associated with the GO term, the GO term level, and *p*-values are indicated.

*Molecular Function*			
**17,20βP versus Control (M_C)**	**Count**	***p*-Value**	**Level**
**Binding**		NS	1
Protein binding	86	0.005	2
Receptor binding	36	0.025	3
Growth factor receptor binding	7	0.015	4
Insulin-like growth factor binding	7	0.012	4
Hormone binding	6	0.033	2
Sulfur compound binding	5	0.047	2
Carbohydrate binding		NS	2
Monosaccharide binding	6	0.020	3
**Catalytic activity**		NS	1
Oxidoreductase activity	32	0.032	2
Oxidoreductase activity, acting on the CH-OH group of donors, NAD or NADP as acceptor	8	0.015	3
Phosphotransferase activity, alcohol group as acceptor	24	0.020	4
Kinase activity	27	0.049	4
Carbohydrate kinase activity	4	0.024	5
Carbohydrate phosphatase activity	3	0.047	6
**Molecular function regulator activity**		NS	1
Growth factor activity	14	0.002	5
**SPH versus Control (S_C)**	**Count**	***p*-Value**	**Level**
**Binding**		NS	1
Protein binding	22	0.044	2
Receptor binding	11	0.047	3
**Molecular function regulatory activity**		NS	1
Cytokine activity	6	0.023	5
**Fresh versus Control (F_C)**	**Count**	***p*-Value**	**Level**
**ATP-dependent activity**		NS	1
RNA helicase activity	5	0.031	3
**Binding**	173	0.030	1
Protein binding	67	<0.001	2
Receptor binding	36	<0.001	3
Cytokine receptor binding	17	<0.001	4
Insulin-like growth factor binding	9	<0.001	4
Carbohydrate derivative binding		NS	2
Lipopolysaccharide binding	3	0.036	3
**Catalytic activity**		NS	1
Disulfide oxidoreductase activity	4	0.015	3
**Molecular function regulator activity**		NS	1
Molecular function activator activity		NS	2
Cytokine activity	14	<0.001	5
Growth factor activity	12	<0.001	6
Hormone activity	10	0.041	6
Enzyme regulator activity		NS	2
Metalloendopeptidase inhibitor activity	3	0.027	6

NS indicates not statistically significant.

## Data Availability

The raw sequencing data used in the manuscript is openly available in the NCBI Sequence Read Archive database (BioProject Accession: PRJNA389564; SRA Accession: SRP108755). Otherwise, the original contributions presented in the study are included in the article/supplementary material; further inquiries can be directed to the corresponding author.

## Supplementary Materials

The following supporting information can be downloaded at: https://www.mdpi.com/article/10.3390/ijms252312683/s1.
